# The Tomato Metallocarboxypeptidase Inhibitor I, which Interacts with a Heavy Metal-Associated Isoprenylated Protein, Is Implicated in Plant Response to Cadmium

**DOI:** 10.3390/molecules25030700

**Published:** 2020-02-06

**Authors:** Anna Manara, Elisa Fasani, Barbara Molesini, Giovanni DalCorso, Federica Pennisi, Tiziana Pandolfini, Antonella Furini

**Affiliations:** Department of Biotechnology, Strada Le Grazie, 15, 37134 Verona, Italy; anna.manara@univr.it (A.M.); elisa.fasani@univr.it (E.F.); barbara.molesini@univr.it (B.M.); giovanni.dalcorso@univr.it (G.D.); federica.pennisi@univr.it (F.P.); tiziana.pandolfini@univr.it (T.P.)

**Keywords:** metallocarboxypeptidase inhibitors, abiotic stress, heavy metals, metal ion binding proteins, *Solanaceae*, *Arabidopsis thaliana*

## Abstract

Metallocarboxypeptidases are metal-dependent enzymes, whose biological activity is regulated by inhibitors directed on the metal-containing active site. Some metallocarboxypeptidase inhibitors are induced under stress conditions and have a role in defense against pests. This paper is aimed at investigating the response of the tomato metallocarboxypeptidase inhibitor (TCMP)-1 to Cd and other abiotic stresses. To this aim, the tomato TCMP-1 was ectopically expressed in the model species *Arabidopsis thaliana*, and a yeast two-hybrid analysis was performed to identify interacting proteins. We demonstrate that *TCMP-1* is responsive to Cd, NaCl, and abscisic acid (ABA) and interacts with the tomato heavy metal-associated isoprenylated plant protein (HIPP)26. *A. thaliana* plants overexpressing *TCMP-1* accumulate lower amount of Cd in shoots, display an increased expression of *AtHIPP26* in comparison with wild-type plants, and are characterized by a modulation in the expression of antioxidant enzymes. Overall, these results suggest a possible role for the TCMP-1/HIPP26 complex in Cd response and compartmentalization.

## 1. Introduction

Protease inhibitors (PIs) are an heterogenous group of molecules widely distributed in different organisms, such as bacteria, fungi, animals, and plants. Among plants, four families (i.e., *Poaceae*, *Fabaceae*, *Solanaceae,* and *Brassicaceae*) account for approximately 66% of all known plant PIs [1]. PIs are crucial components for cellular homeostasis and survival, and participate in several physiological processes, such as the mobilization of storage proteins in seeds, the regulation of endogenous enzymatic activities, and the protection against pests [2,3]. In addition to proteins with higher molecular mass such as serpins, the PI group comprises small/medium range polypeptides (<15 kDa), which include inhibitors of metallocarboxypeptidase and cyclotides [1,4]. Small polypeptide-based PIs are generally composed by a single domain including specific secondary structural elements and disulfide bridges. The first described peptide-based PI, the potato carboxypeptidase inhibitor (PCI; AAC95130), is a 39-residue globular peptide whose structure contains turns, beta strands, and is stabilized by a special disulfide scaffold known as cystine-knot motif [5,6,7], which protects the protein from enzymatic proteolysis, extreme pH, and heat shock. PCIs competitively bind to several target metallocarboxypeptidases (e.g., carboxypeptidase A) in an enzyme-substrate like manner, resulting in the formation of a stable protease-inhibitor complex [8]. Metallocarboxypeptidases are often zinc-dependent peptidases that catalyze the hydrolysis of peptide bonds at the C-terminus of peptides and proteins. PCI inhibition mechanism toward metallocarboxypeptidases depends on two specific motifs in its sequence: the residues 22–30 form an extensive hydrophobic surface allowing contact with carboxypeptidase A, whereas the actual inhibitory segment of the PCI is located at the C-terminal tail [7,9]. After binding, the C-terminal amino acid tail of the PCI enters the active site of carboxypeptidase A. The C-terminal glycine (Gly39) of the PCI is cleaved-off by the protease exposing Val38, whose carboxylate group coordinates with Zn^2+^ in the protease active site, blocking catalytic activity [7,9].

Plant cystine-knot metallocarboxypeptidase inhibitors possess bioactivity also in mammalian cells, since they can target human growth factor receptors either acting as an antagonist of growth factor for receptor binding or by altering their signal transduction pathways [5,10,11,12]. Due to their bioactivity and highly-stable structural scaffold, cystine-knot proteins have received great attention for pharmacological application to develop diagnostic markers and therapeutic agents (for a review see [4]).

Regarding the role of cystine-knot proteins in plants, it was demonstrated that PCIs accumulate in potato tubers and in leaves after wounding [13,14] and after treatment with abscisic acid (ABA) and jasmonate [14]. Consistently with a function in defense responses, the expression of a fusion gene combining a maize PI and PCI conferred resistance to the lepidopteran *Chilo suppressalis* and fungal pathogen *Magnaporthe oryzae*, two detrimental agents affecting rice crops [15]. Metallocarboxypeptidase inhibitors homologous to PCIs were identified in other solanaceous species such as tomato and tobacco. The tomato *TCMP-1* (*Solyc07g007250*) codes for a mature protein of 37 amino acids, displaying 71% identity with the PCI. Its expression is high in flower buds before anthesis, decreases in fertilized flowers, and then slightly increases during fruit growth in green and ripe fruits [11]. Its transcription is upregulated in leaves by wounding, as observed for PCIs, and in response to treatments with systemin, methyl jasmonate, oligogalacturonic acid, and chitosan [16]. A comparative transcriptomic analysis, conducted on *Nicotiana tabacum* leaf trichomes collected from untreated and Cd-treated plants, identified two metallocarboxypeptidase inhibitors (i.e., *NtMCPIa* and *b*, *AB518288* and *AB518289*) that were induced in trichomes of Cd-treated plants [17]. Trichomes contribute to plant stress resistance towards heavy metals by sequestrating and compartmentalizing the toxic ions. In this regard, Harada and collaborators [17] hypothesized that NtMCPI could act as a carrier for the translocation of Cd to tobacco leaf surface.

Cd is one of the most toxic pollutants for plants. It can be absorbed by the roots and transported to the shoots causing various phytotoxic effects, such as wilting, leaf chlorosis, and cell death. Cd is chemically similar to some essential mineral elements (i.e., Ca, Zn, and Fe) and exerts its toxic effect by replacing these elements in key metalloproteins [18]. To cope with Cd stress, plants utilize coordinated strategies responding to the necessity both to directly detoxify the metal and to counteract the cellular stress induced by it. For example, Cd translocation and compartmentalization by means of metal transporters and chelating agents play an important role in metal detoxification [19]. Among metal ligands, heavy metal-associated isoprenylated plant proteins (HIPPs) were only recently taken into consideration. HIPPs are plant-specific metallochaperones characterized by two heavy metal-associated domains and an isoprenylation motif [20]. These proteins are involved in a variety of stresses, possibly playing a role in signal transduction [21,22]. In particular, HIPP26 binds Cd as well as other metals [23] and is associated with Cd tolerance in *Arabidopsis thaliana* [23,24].

Regarding the general mechanisms of stress response, a peculiar role is played by the response to oxidative stress. In the case of heavy metals, reactive oxygen species (ROS) also act as signaling intermediates under metal stress and therefore need to be under tight control by the cell antioxidant system [25]. Moreover, although Cd is not directly involved in redox reactions, it is able to generate oxidative stress through indirect mechanisms, such as alteration of the respiratory and photosynthetic chains and imbalance of anti-oxidative defenses [25,26].

In the present paper, we conducted an in-depth characterization of the *TCMP-1* gene of tomato. We prove that *TCMP-1* expression is induced in tomato leaves by Cd, ABA, and NaCl. By high-throughput yeast two-hybrid (Y2H) screen using the cystine-knot motif containing region of the tomato metallocarboxypeptidase inhibitor (TCMP), we identified the heavy metal-associated isoprenylated protein 26-like (HIPP26; Solyc01g111600) as a putative TCMP interactor. Here, we demonstrate that HIPP26 interacts with TCMP-1 in yeast cells. To gain further insight on the role of TCMP-1, we ectopically expressed it in *A. thaliana,* a species that does not contain cystine-knot metallocarboxypeptidase inhibitor-encoding genes. *TCMP-1* expression determined an altered tolerance to abiotic stresses and a different modulation of the *AtHIPP26* in response to stress in *Arabidopsis*. In addition, in *TCMP-1*-expressing plants, the response to Cd in terms of antioxidative defense and induction of stress-responsive transcripts, differed from that observed in wild-type plants. This suggests that, although solanaceous-specific, TCMP-1 is able to interfere with endogenous stress-related pathways in *Arabidopsis*. *TCMP-1*-overexpressing plants treated with Cd display a reduction in the accumulation of the metal in leaves as compared with wild-type plants. Overall our data support a possible role for TCMP-1 in plant response to heavy metals.

## 2. Results

### 2.1. Cd-Responsive Expression of TCMP-1 in Tomato Leaves

**Figure 1:**
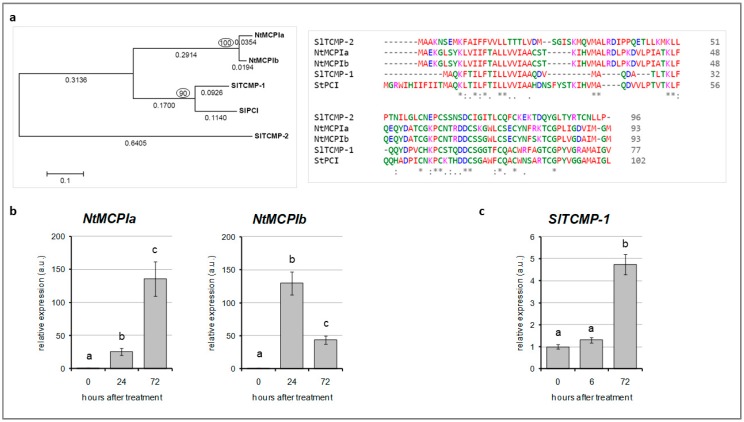
(**a**) Left, evolutionary relationships and protein sequence alignment of 5 well characterized metallocarboxypeptidase inhibitors of *Nicotiana tabacum* (*NtMCPIa* and *NtMCPIb*), *Solanum tuberosum* (StPCI), and *Solanum lycopersicum* (SlTCMP-1 and SlTCMP-2). The evolutionary history was inferred using the neighbor-joining method [27]. The optimal tree with the sum of branch length = 1.67701287 is shown. The percentage of replicate trees in which the associated taxa clustered together in the bootstrap test (100 replicates) is shown next to the branches (values circled). The tree is drawn to scale, with branch lengths (below the branches) in the same units as those of the evolutionary distances used to infer the phylogenetic tree. The evolutionary distances were computed using the Poisson correction method [28] and are in the units of the number of amino acid substitutions per site. The analysis involved 5 amino acid sequences. All positions containing gaps and missing data were eliminated. There were a total of 75 positions in the final dataset. Evolutionary analyses were conducted in MEGA5 [29]. Right, CLUSTAL Omega multiple sequence alignment of the 5 selected proteins [30]. The consensus symbols: “*” identical residues; “:” residues with strongly similar properties; “.” residues with weakly similar properties. (**b**) *NtMCPIa* and *NtMCPIb* mRNA levels in leaves of *Nicotiana tabacum* plants treated with 10 µM CdSO_4_ and collected after 6 and 72 h. (**c**) *SlTCMP-1* expression in leaves collected from tomato plants after 6 and 72 h of treatment with 10 µM CdSO_4_. The values reported in (**b**,**c**) are means ± SE of 3 replicates.

A phylogenetic analysis conducted on five characterized metallocarboxypeptidase inhibitors of *Nicotiana tabacum* (*NtMCPIa* and *NtMCPIb*), *Solanum tuberosum* (StPCI), and *Solanum lycopersicum* (SlTCMP-1 and SlTCMP-2), revealed that *NtMCPIa* and *NtMCPIb* are more closely related to SlTCMP-1 and StPCI, while SlTCMP-2 (Solyc07g049140) constitutes a separate branch (Figure 1a, left). In this regard, SlTCMP-1 protein is 44% and 46% identical to *NtMCPIa* and *NtMCPIb*, respectively (Figure 1a, right).

Considering that *NtMCPIa* and *NtMCPIb* transcripts were found to be differentially expressed in leaf trichomes after Cd treatment [17], we decided to monitor their expression pattern in leaves collected from three-week-old tobacco plants grown in hydroponics and treated with 10 µM CdSO_4_ for 24 and 72 h (Figure 1b). Both genes were strongly upregulated upon Cd stress at the two timepoints. *NtMCPIa* had higher expression after 72 h of treatment, whereas *NtMCPIb* transcript reached its maximum of expression at 24 h of Cd treatment. Due to the high amino acid sequence identity of SlTCMP-1 with NtMCPIs, we decided to analyze the expression of tomato *TCMP-1* in response to Cd. As for tobacco plants, three-week-old tomato plants were treated with 10 µM CdSO_4_. Tomato leaves were collected before the beginning of the treatment (time 0) and at 6 and 72 h after it (Figure 1c). TCMP-1 expression was induced in leaves after 72 h of treatment. Thus, the tomato TCMP-1 is a Cd-responsive gene.

### 2.2. SlTCMP-1 Promoter Is Responsive to Abiotic Stresses

**Figure 2:**
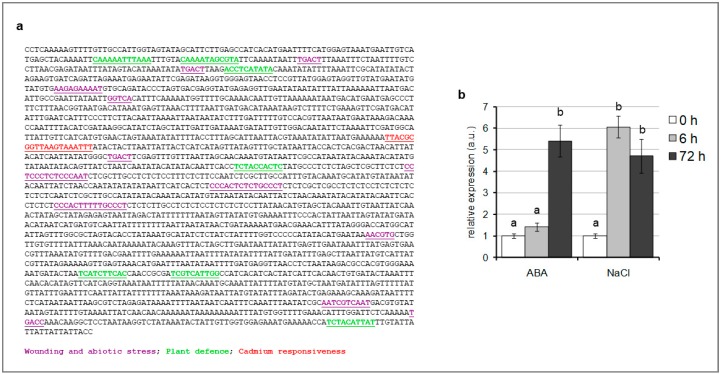
**** (**a**) Distribution of relevant solanaceous-specific cis acting elements predicted in tomato metallocarboxypeptidase inhibitor (TCMP)-1 promoter (plus strand). A 2334 bp-long sequence upstream to the translational start site of *TCMP-1* was analyzed. Cis-acting elements are underlined and presented with different colors. (**b**) *SlTCMP-1* expression in leaves collected from tomato plants after 6 and 72 h of treatment with 50 µM abscisic acid (ABA) and 250 mM NaCl. The values reported are means ± SE of 3 replicates. Statistically significant variation (*p* < 0.05) is marked with different letters.

The altered expression of *TCMP-1* in response to Cd treatment, prompted us to conduct an in silico analysis of the *SlTCMP*-1 promoter regulatory region searching for stress-responsive elements (Figure 2a). A 2334 bp-long sequence upstream of the ATG translation initiation codon of *TCMP-1* was chosen as putative promoter sequence (Figure 2a). By using the Plant Promoter Analysis Navigator (PlantPAN3.0; [31]), a cis element starting at position 795 of the plus DNA strand implicated in Cd responsiveness was found (Figure 2a), which corroborates the results on the TCMP-1 gene expression regulation by Cd (Figure 1c). In addition, several cis elements related to wounding and abiotic stress as well as to plant defense were also predicted (Figure 2a). Given this indication, we analyzed *TCMP-1* expression in tomato plantlets after imposing various stress treatments, using ABA, which acts as a mediator in plant responses to a range of stresses, and NaCl (Figure 2b). Treatment with 50 µM ABA induced *TCMP-1* expression after 72 h, whereas salt treatment (250 mM NaCl) produced an upregulation after both 6 and 72 h (Figure 2b).

### 2.3. The Tomato HIPP26, A Heavy Metal-Associated Isoprenylated Protein 26-like, Interacts with TCMP-1 In Vivo in Yeast

**Figure 3:**
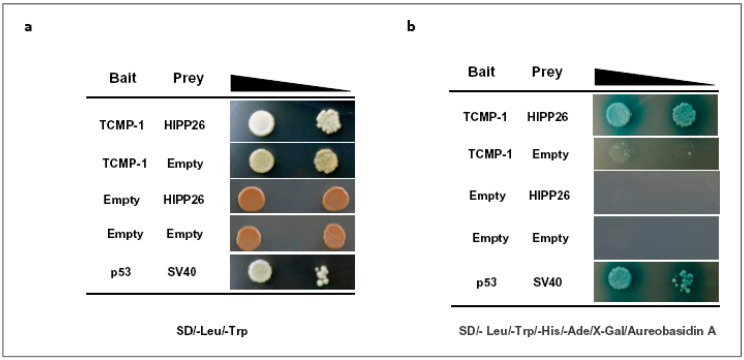
Yeast-two hybrid analysis of TCMP-1 interaction with heavy metal-associated isoprenylated plant protein 26-like (SlHIPP26). Yeast cells transformed with different combinations of constructs containing TCMP-1 fused with the DNA binding domain (BD) (bait; TCMP-1), HIPP26 fused with the activation domain (AD) (prey; HIPP26), the BD alone (bait; empty), and the AD alone (prey; empty) were grown on two different selective media. Interaction of p53 with SV40 was used as a positive control of the mating system. (**a**) The mated cultures were spotted on SD/-Leu/-Trp control plates to confirm the presence of both plasmids in yeast cultures. (**b**) The mated cultures were spotted on synthetic quadruple dropout agar plates (SD/-Leu/-Trp/-His/-Ade/X-Gal/Aureobasidin A) to check genuine positive interactions. Positive interaction was confirmed by the growth of blue colonies (◣, indicates that two increasing dilutions of the mated cultures were used).

To investigate the role of TCMPs in tomato, we conducted a high-throughput yeast two-hybrid (Y2H) screen using the cystine-knot motif containing region of TCMP as bait to search for interacting partners (our unpublished data). Among the putative partners, we identified an interactor protein (Solyc01g111600.2) which codes for heavy metal-associated isoprenylated plant protein 26-like (hereafter indicated as SlHIPP26). The region responsible for the interaction with the bait covers the entire SlHIPP26 153-amino acid long sequence. To test whether SlHIPP26 physically interacts with TCMP-1, a direct GAL4-based two-hybrid in vivo assay was conducted. The nucleotide sequence containing the cystine-knot motif and corresponding to the mature 37 amino acid-long TCMP-1 peptide representing the bait was expressed as a fusion to the GAL4 DNA-binding domain (BD) (pGBKT7-BD-TCMP-1 recombinant vector) (Figure 3). As prey we used the entire protein coding sequence of SlHIPP26 which was fused in frame to the GAL4-activation domain (AD) (pGADT7-AD-SlHIPP26) (Figure 3). As shown in Figure 3, TCMP-1 and SlHIPP26 proteins interact in yeast cells (Figure 3).

Using the TomExpress RNA-Seq platform [32], we found that SlHIPP26, which displays 92% sequence similarity with HIPP26 of *Arabidopsis* (Figure S1), is ubiquitously expressed in tomato, with leaves and meristems being the organs that display the highest levels of expression (Figure S2). It is interesting to note that after Cd treatment, *TCMP-1* expression is induced in the leaves, where its partner is highly expressed.

### 2.4. SlTCMP-1 Overexpression in Arabidopsis thaliana Induces A Different Modulation of HIPP26 in Response to Stress

To better define the role of *TCMP-1* gene, we decided to produce *TCMP-1* expressing lines in *Arabidopsis*, a species that lacks cystine-knot carboxypeptidase inhibitor-encoding genes. We placed the coding region of *TCMP-1* gene behind the cauliflower mosaic virus (CaMV) 35S promoter and transformed it into *Arabidopsis* by floral dipping (Figure S3, upper). Two lines (i.e., *35S::TCMP-1#A* and *35S::TCMP-1*#B), expressing the transgene (Figure S3, lower) were selected for further analysis.

**Figure 4:**
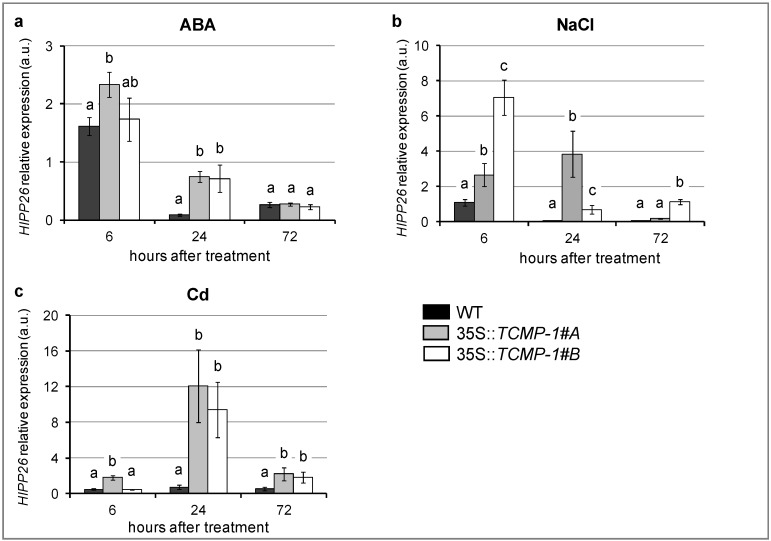
Real-time polymerase chain reaction (PCR) analysis of *AtHIPP26* transcript in wild-type (WT) and *TCMP-1* overexpressing *A. thaliana* plants treated for 6, 24, and 72 h with (**a**) 50 µM ABA, (**b**) 250 mM NaCl, and (**c**) 10 µM CdSO_4_. The histograms show the 2^−ΔΔCT^ values ± SE (*n* = 3). Each value reported in histograms is normalized to the same sample in control conditions at the same time point. Statistically significant variations (*p* < 0.05) are marked with different letters.

The expression of the transgene did not determine any visible morphological alteration in the transgenic lines, which appeared indistinguishable from wild-type (WT) plants (data not shown). To evaluate if *TCMP-1* expression modifies the *AtHIPP26* transcription in response to different abiotic stresses, the expression of *AtHIPP26* gene was analyzed in leaves after treatment with ABA, NaCl, and CdSO_4_. In control conditions, the *TCMP-1* overexpression in *A. thaliana* did not alter *HIPP26* expression levels (data not shown). Differently, in abiotic stress conditions, the presence of *TCMP*-*1* induced a modulation of *HIPP26* expression in *A. thaliana*. After one day of exposure to 50 μM ABA, *AtHIPP26* transcript level was higher in *TCMP-1* overexpressing lines in respect to wild-type (Figure 4a). Similarly, *AtHIPP26* expression levels were higher in both overexpressing plants than in wild-type after 6 and 24 h of 250 mM NaCl treatment (Figure 4b). Exposure to 10 µM CdSO_4_, a concentration that induces a moderate stress [33], for 24 and 72 h induced a higher *AtHIPP26* expression in *TCMP*-*1*-overexpressing plants in respect to wild-type (Figure 4c). Overall these results demonstrate that *TCMP-1* overexpression enhanced the response of *HIPP26* to stress conditions.

### 2.5. Cd Treatment Influences Oxidative Stress Response and Metal Accumulation in A. thaliana Plants Overexpressing SlTCMP-1

**Figure 5:**
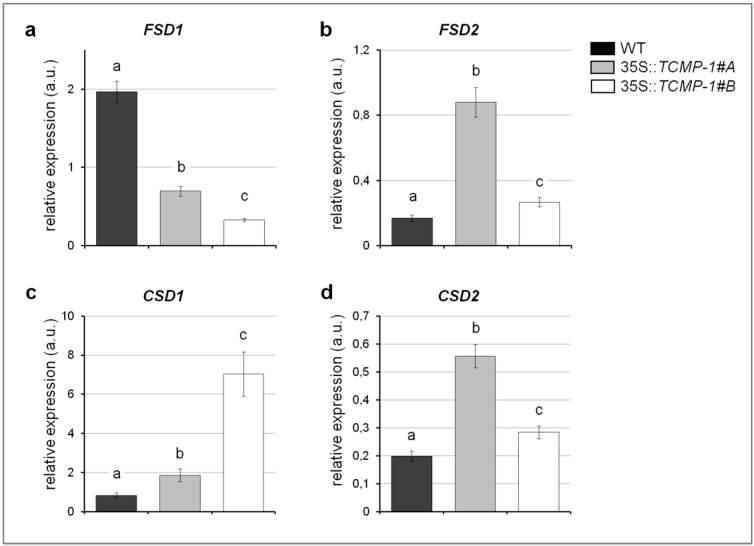
Real-time PCR analysis of (**a**) *FSD1*, (**b**) *FSD2*, (**c**) *CSD1,* and (**d**) *CSD2* transcripts in wild-type (WT) and *TCMP-1*-overexpressing plants treated for 24 h with 10 µM CdSO_4_. The histograms show the 2^−ΔΔCT^ values ± SE (*n* = 3). Each value reported in histograms is normalized to the same sample in control conditions at the same time point. Statistically significant variations (*p* < 0.05) are marked with different letters.

Heavy metal stress conditions produce reactive oxygen species (ROS) in plants causing oxidative stress damage and, at the same time, ROS play a signaling role in plants adaptation to stress [34]. As reported by Smeets et al. [33], Cd treatment induced an alteration of transcript abundance of several genes involved in plant antioxidant machinery in *A. thaliana*. After Cd treatment a significant increase in the activity of both Fe superoxide dismutase (FSD) and ascorbate peroxidase (APX) was observed, while Cu/Zn superoxide dismutase (CSD) decreased [33] probably due to a modulation of enzyme biosynthesis at the transcriptional level [35]. To test the sensitivity of *TCMP-1*-overexpressing lines to ROS, we analyzed the expression of genes coding for enzymes involved in ROS detoxification in Cd-treated plants (Figure 5).

**Figure 6:**
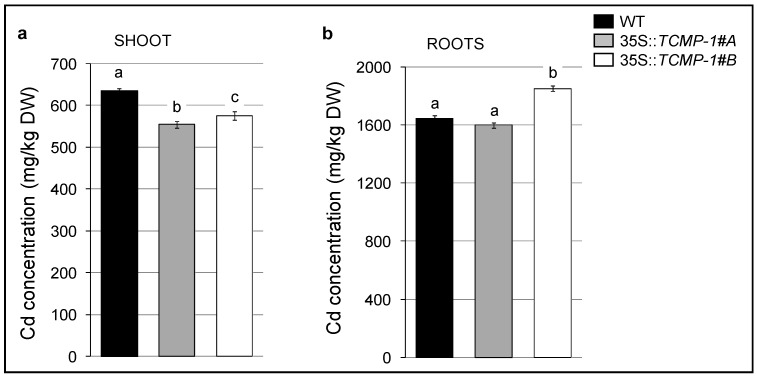
Cd accumulation in shoots (**a**) and roots (**b**) of wild-type (WT) and *TCMP-1*-overexpressing plants grown for 10 days in hydroponic solution containing 10 µM CdSO_4_. Each value represents the mean ± SE (*n* = 3). Statistically significant variations (*p* < 0.05) are marked with different letters.

When plants were grown on medium supplemented with 10 µM CdSO_4_ for 10 days, the expression of *FSD1* was downregulated in *TCMP-1*-overexpressing plants, whereas the expression of *CSD1* and *CSD2* was upregulated in comparison to that in wild-type (Figure 5a,c,d). Furthermore, an increase expression in the levels of the plastidial *FSD2* was observed in both *TCMP-1*-overexpressing lines although more marked in *35S::TCMP-1#A* line (Figure 5b). Collectively, expression of superoxide dismutase (SOD) genes in transgenic plants is significantly perturbed in comparison with wild-type. Moreover, the effect of Cd treatment was investigated by measuring the superoxide anion radical (O^2−^) in leaves using the nitroblue tetrazolium (NBT) staining method. After treatment with 10 µM CdSO_4_ for 10 days, no visible differences in O^2−^ accumulation were observed (Figure S4b). Cd accumulation was measured in the shoots of plants treated in a hydroponic solution containing 10 µM CdSO_4_. After 10 days under this treatment regimen, *35S::TCMP-1* transgenic shoots remained phenotypically indistinguishable from control plants (Figure S4a). However, Cd content in the shoots of *35S::TCMP-1* lines was significantly lower (approximately 10%) than that in wild-type shoots (Figure 6a). Thus, the expression of the transgene resulted in a lower Cd accumulation in shoot. On the other hand, in roots, Cd accumulation is different between the two overexpressing lines, with line 35S::*TCMP-1#A* accumulating Cd levels similar to wild-type, and 35S::*TCMP-1#B* showing higher Cd content (Figure 6b).

### 2.6. SlTCMP-1 Overexpression in A. thaliana Affects Germination Rate under Stress Condition

**Figure 7:**
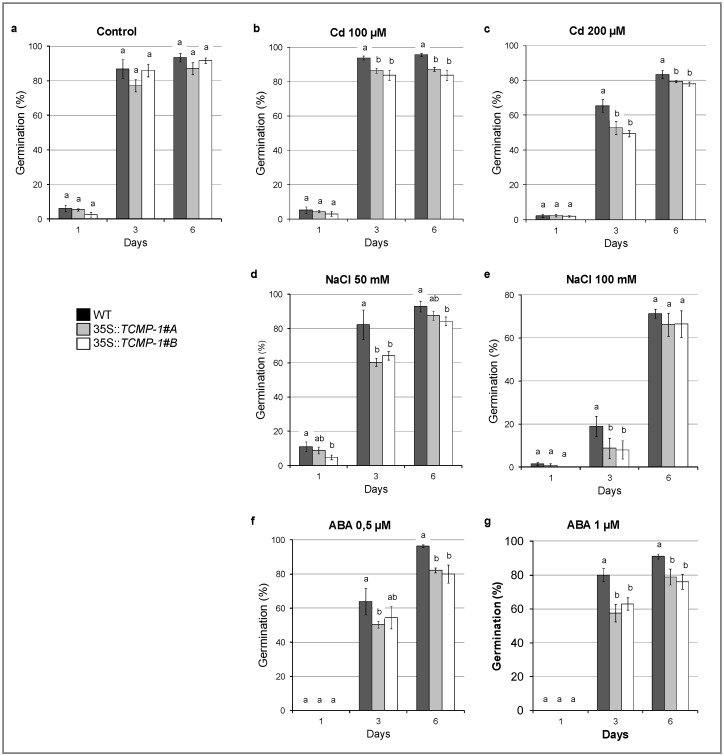
Effect of NaCl, ABA, and Cd on wild-type (WT) and *TCMP-1-*overexpressing line germination. (**a**) Germination in control condition. Effect of (**b**) 100 and (**c**) 200 µM CdSO_4_, (**d**) 50 and (**e**) 100 µM NaCl, and (**f**) 0.5 and (**g**) 1 µM ABA exposure for 1, 3, and 6 days after sowing. Each value represents the mean ± SE. Approximately 100 seeds from each genotype were analyzed in three independent experiments. Statistically significant variations (*p* < 0.05) are marked with different letters.

Considering that abiotic stress responsive elements were found in the promoter of *SlTCMP-1* (Figure 2a), the effect of *SlTCMP-1* overexpression was analyzed in *A. thaliana* in response to different abiotic stresses. To investigate the tolerance to abiotic stress of wild-type and transgenic lines, different treatments of Cd, NaCl, and ABA were performed on Murashige and Skoog (MS) medium to determine the germination rates (Figure 7). For control condition and NaCl treatment, seedlings were considered as germinated when they produced green cotyledons, while in the presence of Cd and ABA, germination was determined by radical emergence (Figure 7). Under control conditions no differences were observed in germination of wild-type and *TCMP-1*-overexpressing plants (Figure 7a). Cd exposure at 100 and 200 µM reduced the germination of overexpressing plants after 3 and 6 days of treatment as compared to Cd-treated wild-type seeds (Figure 7b,c). Similarly, after 3 days of exposure to 50 and 100 mM NaCl, the percentage of germinating plants was significantly lower in the *TCMP-1*-overexpressing lines compared to wild-type plants. However, after 6 days of exposure to NaCl, overexpressing plants showed a recovery in germination capacity (Figure 7d,e). The germination capacity was also tested adding ABA to the medium considering radical emergence. Treatment with 0.5 and 1 µM ABA for 3 and 6 days reduced the germinability of overexpressing plants in comparison with wild-type (Figure 7f,g). Thus, the ectopic expression of *TCMP-1* in *Arabidopsis* determined an increased sensitivity to stress and Cd treatment.

## 3. Discussion

This study was designed to gain insights on the role of tomato metallocarboxypeptidase inhibitor (TCMP-1) in response to stress conditions. Similar to its homolog in potato, this protease inhibitor is induced in leaves by wounding and by elicitors of responses to biotic stress [13,14]. These experimental evidences are in accordance with a role for this protein in defense against pests [15]. Our study demonstrates that *TCMP-1* is also responsive to abiotic stress, such as saline stress and Cd toxicity.

Considering a possible biotechnological application, we tried to express *SlTCMP-1* in *Arabidopsis*, with the aim of understanding its contribution to metal stress response. Indeed, this protein, and its biological context, is not present in this model species since no homolog to *TCMP-1* is detected in the *Arabidopsis* genome. The ectopic expression of *SlTCMP-1* in *Arabidopsis* does not alter plant development and no visible phenotype was observed under standard growth conditions. When exposed to Cd stress, even if no overall difference was observed in the amount of O^2−^ accumulation, *A. thaliana TCMP-1* overexpressing plants accumulate less Cd in shoots, in comparison to wild-type plants. Even if it is not considered a redox-reactive metal, since it does not react with H_2_O_2_ to generate reactive oxygen species via Fenton reaction, Cd can nevertheless induce oxidative stress by depleting antioxidant thiol-containing compounds and enzymes or altering the distribution and homeostasis of other metals, including the redox active ones [36]. Among the enzymes involved in ROS detoxification, superoxide dismutase (SOD) is responsible for the dismutation of superoxide to hydrogen peroxide [37]. In the *A. thaliana* genome, seven SOD-coding genes are present: three FeSOD (*FSD1*, *FSD2,* and *FSD3* isoforms), three Cu/ZnSODs (*CSD1*, *CSD2,* and *CSD3* isoforms) and one MnSOD (*MSD1*). SOD expression is modulated in response to various stresses that induce ROS accumulation [33]. Alteration of the cellular oxidative status upon Cd treatment induced an increase in *FSD1* and a reduction in *CSD2* expression [33]. Moreover, Cd induced an increase in the activity of FeSOD, while it reduced CuZnSOD activity in *A. thaliana*; the observed alteration of SOD activity is probably due to a different enzyme biosynthesis controlled at the translational or transcriptional level [35]. Upon Cd treatment, *A. thaliana* plants overexpressing *SlTCMP-1* showed a reduced *FSD1* expression and an increase in *CSD1* and *CSD2* expression levels in comparison to wild-type (this work), which could be correlated with the lower Cd content in leaves. The opposite effect on the expression of plastidial *FSD2* and cytosolic *FSD1* was probably due to the different subcellular localization of the two proteins.

Since *SlTCMP-1* is responsive to abiotic stress, we analyzed the effects of Cd, ABA, and NaCl treatments on germination of transgenic *Arabidopsis* plants. All three treatments delayed seed germination in both wild-type and transgenic plants, with the latter being more sensitive to the stress imposed. It is known that Cd inhibits seed germination through a variety of mechanisms, in particular by misbalancing the ROS content which is, in turn, highly correlated to ABA signaling. Indeed, ROS are thought to modulate hormonal interactions, by stimulating Gibberellin (GA) biosynthesis and inducing ABA catabolism (reviewed in [38]). However, the relationship between ROS and hormone signaling in controlling seed germination is still under debate. In addition, the osmotic stress applied as NaCl treatment inhibits seed germination in transgenic plants to a greater extent than wild-type. Salt stress induces cellular ROS imbalance, being strictly interconnected with ABA signaling through an ABA-dependent pathway; for instance, tobacco plants overexpressing ABA-induced transcription factor WRKY17 from cotton showed enhanced germination sensitivity to salt stress and ABA treatment and lower expression of ROS-scavenging genes [39].

By Y2H screen, this study proves that SlTCMP-1 interacts with the metal-ion binding protein SlHIPP26. SlHIPP26 is highly similar to the *Arabidops* is HIPP26 (also known as AtFP6), whose expression is induced by abiotic stress, such as cold and saline conditions, and is differentially modulated by heavy metal application. Indeed, Cd and Zn ions induce *AtHIPP26* expression, while Cu and Pb do not enhance *AtHIPP26* expression [23]. Overexpression of *AtHIPP26* is able to induce Cd tolerance in transgenic plants [40]. Localization experiments showed a putative dual localization: (i) at the plasma membrane, where it putatively interacts with acyl-CoA-binding protein 2 (ACBP2) and could be involved in mitigating lipid peroxidation due to Cd stress [23]; and (ii) in the nucleus, AtHIPP26 harboring a nuclear localization signal, where it could interact with transcription factors, such as AtHB29 and AtHB21, modulating gene packages involved in tolerance to Cd stress [40]. Moreover, AtHIPP26 is able (at least in vitro) to bind metals such as Cd, Cu, and Pb [40] and seems to be specifically expressed in vascular tissues. In *Nicotiana benthamiana*, HIPP26 proteins also seem to bind to the potato mop-top virus movement protein TGB1; by a still unknown mechanism, such interaction is responsible for a release of membrane-associated NbHIPP26, and its redirection via microtubules to the nucleus. In this last compartment, the TGB1-HIPP26 complex activates drought stress response, facilitating virus long-distance movement [41]. Therefore, by binding to a variety of stress-related molecules (i.e., movement proteins, heavy metals) *NbHIPP26* can be considered a sensor which acts as a plasma membrane-to-nucleus signal. It is worth noting that upon abiotic stress conditions, endogenous *AtHIPP26* is responsive to heterologous expression of *SlTCMP-1*, even though *A. thaliana* lacks genes homologous to *SlTCMP-1*. In fact, in our *TCMP-1* overexpressing plants, *AtHIPP26* expression is induced by ABA, NaCl, and Cd to a greater extent than in wild-type. Considering that HIPP26 plays a role in the signaling pathway between perception of stress and modulation of target gene expression, the increased amount of HIPP26 and the presence of the interactor SlTCMP-1 might be responsible for the altered modulation of ROS scavenging genes in shoots. It would be interesting to evaluate whether these effects are attenuated when TCMP-1 is overexpressed in a *hipp26* mutant background.

As previously mentioned, *TCMP-1* overexpressing *Arabidopsis* plants accumulate lower amounts of Cd in shoots and show an enhanced expression of *AtHIPP26* upon Cd stress. Regarding this last result, it must be recalled that AtHIPP26 is able to bind Cd and, on the other hand, SlTCMP-1 is ectopically expressed also in the roots of transgenic plants. Therefore, it would be reasonable to hypothesize that Cd root-to-shoot translocation may be impaired in transgenic plants. Indeed, in the 35S::*TCMP-1#B* overexpressing line, Cd content in roots increases in comparison to wild-type and 35S::*TCMP-1#A*, but Cd content in shoots is similar between the two transgenic lines pointing to a probable effect of SlTCMP1-HIPP16 interaction on root-to-shoot Cd translocation. Cd could either be “immobilized” by over-accumulating HIPP26 itself, or by the heterologous complex HIPP26-SlTCMP-1 which could be involved in accumulation of Cd in the apoplast of root cells, as previously suggested [17].

In conclusion, this work highlights that a new mechanism may be shifted from one species to another, with similar molecular details, but giving different results. Indeed, if *A. thaliana* plants overexpressing *AtHIPP26* alone are more tolerant to Cd [23], it seems that *AtHIPP26* induction driven by *SlTCMP-1* overexpression is associated with Cd sensitivity, at least considering seed germination, and a reduced amount of Cd translocated to the above-ground tissues. Further studies are needed to unravel the molecular pathways downstream of the HIPP26-TCMP-1 complex formation. The biotechnological exploitation of such mechanism can be evaluated in phytoremediation approaches, considering metal phytostabilization in the root compartment rather than in its accumulation in aerial tissues.

## 4. Materials and Methods

### 4.1. Plant Genotypes, Growth Conditions, and Treatments

Wild-type tomato (*Solanum lycopersicum* cv. L276) and tobacco (*Nicotiana tabacum* cv. Petit Havana SR1) were used for the expression analysis of the metallocarboxypeptidase inhibitors *TCMP-1* (*Solyc07g007250*) and *NtMCPIa/NtMCPIb* (*AB518288.1/AB518289.1*), respectively. Wild-type and transgenic *Arabidopsis thaliana* plants (ecotype Columbia, Col-0) were tested for stress tolerance, Cd accumulation, and expression of stress-related genes. For treatments in hydroponics conditions, seeds were sterilized and sown on solid MS medium [42] supplemented with 30 g/L sucrose and vernalized for 2 d at 4 °C. Plants were grown in half-strength Hoagland’s solution [43] for two weeks in a controlled growth chamber (16 h photoperiod at 23 °C). Plants were then treated with 10 µM CdSO_4_ (for all plant species considered), 250 mM NaCl, or 50 µM abscisic acid (ABA) (for tomato and *Arabidopsis*); the control condition was obtained by maintaining plants in standard half-strength Hoagland’s solution. For the analysis of gene expression, leaves were collected at different times (e.g., 6, 24, and 72 h) after treatment. For the analysis of superoxide anion (O^2−^) and the quantification of Cd accumulation, leaves were collected after 10 days from the beginning of Cd treatment. Leaves were homogeneous in age, size, and exposition to light; leaves were collected in triplicate from three different plants for each analysis.

### 4.2. Yeast Two-Hybrid Assay

To check the interaction between *TCMP-1* and the heavy metal-associated isoprenylated plant protein 26-like (Solyc01g111600.2.1; hereafter referred to as HIPP26), the Matchmaker Gold Yeast Two-Hybrid System (Clontech, Saint-Germain-en-Laye, France) was used following the manufacturer’s instruction with slight modifications [44,45]. The coding region corresponding to the 37 amino acid-long mature portion of TCMP-1 protein was amplified using the primers reported in Table S1 (Supplementary Materials), cloned in frame in the double digested (BamHI/EcoRI) pGBKT7-BD vector and then the recombinant vector introduced into Y2HGold yeast strain. Similarly, the coding region of *Solyc01g111600.2.1* corresponding to the entire protein was amplified (see primer pairs listed in Table S1), cloned in frame into the double digested (BamHI/EcoRI) pGADT7-AD vector, and finally introduced into Y187 yeast strain. Both recombinant plasmids were checked by sequencing before performing yeast mating. To verify the bait–prey interaction, a single colony of the Y2HGold yeast strain harboring the pGBKT7 vector for DNA-BD-TCMP-1 expression was mated with a single colony of Y187 yeast strain containing pGADT7 vector for AD-HIPP26 expression. The mated culture was spread on agar plates containing the selective recommended media followed by incubation at 30 °C for 3 days. Single colonies were spotted on SD/-Leu/-Trp (to check for the presence of both recombinant plasmids) and Quadruple dropout medium supplemented with X-alpha-gal and Aureobasidin A (QDO/X/A) (SD/-Leu/-Trp/-His/-Ade/X-Gal/Aureobasidin A) plates, the last medium represents the most stringent growth condition for the tested interaction, followed by incubation at 30 °C for 3 days.

### 4.3. Genetic Transformation of Arabidopsis Plants Expressing the Tomato TCMP-1 Gene

*TCMP-1* expressing lines were generated in *A. thaliana* plants by *Agrobacterium*-mediated floral dipping transformation [46]. The pCAMBIA 1200 binary vector harbors in the T-DNA the hygromycin resistance gene and a genetic cassette containing a 234 nucleotide-long fragment corresponding to the *TCMP-1* coding sequence under the control of the CaMV 35S promoter (*35S::TCMP-1*) and of the nopaline synthase gene terminator (*nos*). Transgenic plants were selected on MS agar plates (2.15 g/L MS salts, 0.8% plant agar (*w*/*v*), 1% sucrose, pH 5.7) supplemented with 13 mg/L hygromycin B. The expression of *TCMP-1* coding sequence was checked by semiquantitative real-time polymerase chain reaction (RT-PCR) analysis using primers reported in Table S1.

### 4.4. Analysis of Gene Expression

Total RNA was isolated with TRIzol Reagent (Thermo Fisher Scientific, Waltham, MA, USA); three pools from leaves were used as biological replicates. After DNase treatment, first-strand cDNA was synthesized using the Superscript II Reverse Transcriptase Kit (Thermo Fisher Scientific). Real-time reverse transcription polymerase chain reaction (RT-PCR) was performed with a StepOnePlus Real-Time PCR System (Thermo Fisher Scientific, Waltham, MA, USA) using Luna^®^ Universal qPCR Master Mix (New England Biolabs, Ipswich, MA, USA). Each reaction was carried out for 40 amplification cycles, in triplicate using the primers reported in Table S1. The amplification efficiency of each primer pair was confirmed using LinRegPCR v.7.5 software [47]. Data were normalized using the endogenous reference genes *SlActin* (*Solyc11g005330.2.1*) for tomato, *NtActin* (*XM_016628756.1*) for tobacco, and *Ubiquitin10* for *Arabidopsis thaliana* (*At4g05320*). Data analysis was performed using the 2^−ΔΔCT^ method [48], comparing expression in each treated sample with the relative control collected at the same time.

### 4.5. In Vitro Analysis of Stress Tolerance on 35S::TCMP-1 Arabidopsis Plants

For germination analysis, *Arabidopsis* WT and transgenic seeds were sterilized and sown on plates containing MS medium supplemented with 1% sucrose and different concentrations of ABA (0.5 and 1.0 µM), CdSO_4_ (100 and 200 µM), and NaCl (50 mM and 100 mM) for 1, 3, and 6 days. Plates were placed in the phytochamber under controlled conditions (16 h light/8 h dark, illumination 100–120 μmol/m^2^/s, day/night temperature 22 °C/18 °C). Approximately 100 seeds of each genotype were sown on each plate and scored for germination considering the emersion of roots. For NaCl treatment, germination was scored by cotyledons emersion. Each experiment was performed in triplicate.

### 4.6. Analysis of Superoxide Anion in Cd-Treated 35S::TCMP-1 Arabidopsis Plants

After 10 days of treatment with 10 µM CdSO_4_ in hydroponics, detection of O^2˙^¯ was performed by treating leaves with nitroblue tetrazolium (NBT) as described in [49]. Briefly, leaves were detached from plants and vacuum-infiltrated with 10 mM potassium phosphate buffer pH 7.8, containing 10 mM NaN_3_, and then incubated in 0.1% NBT in 10 mM potassium phosphate buffer pH 7.8 for 1 h at room temperature. After the incubation, leaves were cleared by boiling in acetic acid:glycerol:ethanol (1:1:3 *v*/*v*/*v*) solution and then stored in a glycerol:ethanol (1:4 *v*/*v*) solution until photographs were taken. O^2˙^¯ was visualized as blue spots produced by NBT reduction to formazan.

### 4.7. Analysis of Cd Accumulation of 35S::TCMP-1 Arabidopsis Plants

After 10 days of treatment with 10 µM CdSO_4_ in hydroponics, shoot and root apparatuses of wild-type and *35S::TCMP-1 Arabidopsis* plants were oven-dried at 60 °C and subjected to microwave-assisted acid digestion (EPA 3050, AOAC 17th ED. 2000; 999.10). The Cd determination was conducted with Inductively Coupled Plasma Optical Emission Spectroscopy (ICP-OES), SPECTRO ARCOS (SPECTRO Analytical Instruments GmbH, Kleve, Germany; AOAC Official Method 985.01 20th Edition). Calibration standards were matched with 1% absolute ethanol (Prolabo VWR International PBI S.r.l., Milan, Italy). The concentration range of the calibration solution was between 0 and 100 mg/L.

### 4.8. Statistical Analysis

Statistical analysis was performed using GraphPad Prism version 5 software. Multiple variables were analyzed using one-way ANOVA followed by Tukey’s post hoc test. In histograms, data are represented as means ± SE of 3 replicates. Statistically significant variations (*p* < 0.05) are marked with different letters.

## Supplementary Materials

The following are available online, Figure S1: Sequence alignment of HIPP26 proteins of tomato and *Arabidopsis* (At4G38580) obtained by CLUSTAL Omega [30], Figure S2: Expression of *SlHIPP26* gene in organs of various *S. lycopersicum* cultivars, Figure S3: Schematic drawing of *35S::TCMP-1* genetic construct and RT-PCR analysis of *TCMP-1* gene in wild-type and transgenic *Arabidopsis thaliana* plants, Figure S4: Effects of Cd treatment on WT, *35S::TCMP-1#A* and *#B* plants after 10 days of hydroponic cultivation, Table S1: List of primers used in this work.

## References

[1] Hellinger R., Gruber C.W. (2019). Peptide-based protease inhibitors from plants. Drug Discov. Today.

[2] Rawlings N.D., Tolle D.P., Barrett A.J. (2004). Evolutionary families of peptidase inhibitors. Biochem. J..

[3] Clemente M., Corigliano M.G., Pariani S.A., Sánchez-López E.F., Sander V.A., Ramos-Duarte V.A. (2019). Plant serine protease inhibitors: Biotechnology application in agriculture and molecular farming. Int. J. Mol. Sci..

[4] Molesini B., Treggiari D., Dalbeni A., Minuz P., Pandolfini T. (2017). Plant cystine-knot peptides: Pharmacological perspectives. Br. J. Clin. Pharmacol..

[5] Blanco-Aparicio C., Molina M.A., Fernandez-Salas E., Frazier M.L., Mas J.M., Querol E., Avilés F.X., de Llorens R. (1998). Potato carboxypeptidase inhibitor, a T-knot protein, is an epidermal growth factor antagonist that inhibits tumor cell growth. J. Biol. Chem..

[6] Craik D.J. (2012). Host-defense activities of cyclotides. Toxins.

[7] González C., Neira J.L., Ventura S., Bronsoms S., Rico M., Avilés F.X. (2003). Structure and dynamics of the potato carboxypeptidase inhibitor by 1H and 15N NMR. Proteins.

[8] Ryan C.A., Hass G.M., Kuhn R.W. (1974). Purification and properties of a carboxypeptidase inhibitor from potatoes. J. Biol. Chem..

[9] Rees D.C., Lipscomb W.N. (1982). Refined crystal structure of the potato inhibitor complex of carboxypeptidase A at 2.5 A resolution. J. Mol. Biol..

[10] Sitja-Arnaud M., Molina M.A., Blanco-Aparicio C., Ferrer-Soler L., Lorenzo J., Aviles F.X., Querol E., de Llorens R. (2005). Mechanism of action of potato carboxypeptidase inhibitor (PCI) as an EGF blocker. Cancer Lett..

[11] Cavallini C., Trettene M., Degan M., Delva P., Molesini B., Minuz P., Pandolfini T. (2011). Antiangiogenic effects of two cystine-knot miniproteins from tomato fruit. Br. J. Pharmacol..

[12] Treggiari D., Zoccatelli G., Molesini B., Degan M., Rotino G.L., Sala T., Cavallini C., MacRae C.A., Minuz P., Pandolfini T. (2015). A cystine-knot miniprotein from tomato fruit inhibits endothelial cell migration and angiogenesis by affecting vascular endothelial growth factor receptor (VEGFR) activation and nitric oxide production. Mol. Nutr. Food Res..

[13] Graham J.S., Ryan C.A. (1981). Accumulation of a metallocarboxypeptidase inhibitor in leaves of wounded potato plants. Biochem. Biophys. Res. Commun..

[14] Villanueva J., Canals F., Prat S., Ludevid D., Querol E., Aviles F.X. (1998). Characterization of the wound-induced metallocarboxypeptidase inhibitor from potato. cDNA sequence, induction of gene expression, subcellular immunolocalization and potential roles of the C-terminal propeptide. FEBS Lett..

[15] Quilis J., López-García B., Meynard D., Guiderdoni E., San Segundo B. (2014). Inducible expression of a fusion gene encoding two proteinase inhibitors leads to insect and pathogen resistance in transgenic rice. Plant Biotechnol. J..

[16] Díez-Díaz M., Conejero V., Rodrigo I., Pearce G., Ryan C.A. (2004). Isolation and characterization of wound-inducible carboxypeptidase inhibitor from tomato leaves. Phytochemistry.

[17] Harada E., Kim J.-A., Meyer A.J., Hell R., Clemens S., Choi Y.-E. (2010). Expression Profiling of Tobacco Leaf Trichomes Identifies Genes for Biotic and Abiotic Stresses. Plant Cell Physiol..

[18] DalCorso G., Farinati S., Maistri S., Furini A. (2008). How plants cope with cadmium: Staking all on metabolism and gene expression. J. Integr. Plant Biol..

[19] Mendoza-Cózatl D.G., Jobe T.O., Hauser F., Schroeder J.I. (2011). Long-distance transport, vacuolar sequestration, tolerance, and transcriptional responses induced by cadmium and arsenic. Curr. Opin. Plant Biol..

[20] de Abreu-Neto J.B., Turchetto-Zolet A.C., de Oliveira L.F.V., Bodanese Zanettini M.H., Margis-Pinheiro M. (2013). Heavy metal-associated isoprenylated plant protein (HIPP): Characterization of a family of proteins exclusive to plants. FEBS J..

[21] Barth O., Zschiesche W., Siersleben S., Humbeck K. (2004). Isolation of a novel barley cDNA encoding a nuclear protein involved in stress response and leaf senescence. Physiol. Plant..

[22] Tehseen M., Cairns N., Sherson S., Cobbett C.S. (2010). Metallochaperone-like genes in *Arabidopsis thaliana*. Metallomics.

[23] Gao W., Xiao S., Li H.Y., Tsao S.W., Chye M.L. (2009). *Arabidopsis thaliana* acyl-CoA-binding protein ACBP2 interacts with heavy-metal-binding farnesylated protein AtFP6. New Phytol..

[24] Gao W., Li H.Y., Xiao S., Chye M.L. (2010). Protein interactors of acyl-CoA-binding protein ACBP2 mediate cadmium tolerance in Arabidopsis. Plant Signal. Behav..

[25] Sandalio L.M., Rodríguez-Serrano M., Gupta D.K., Archilla A., Romero-Puertas M.C., Luis A., Ahmad P., Prasad M.N.V. (2012). Reactive oxygen species and nitric oxide in plants under cadmium stress: From toxicity to signaling. Environmental Adaptations and Stress Tolerance of Plants in the Era of Climate Change.

[26] Cuypers A., Plusquin M., Remans T., Jozefczak M., Keunen E., Gielen H., Opdenakker K., Ravindran Nair A., Munters E., Artois T.J. (2010). Cadmium stress: An oxidative challenge. Biometals.

[27] Saitou N., Nei M. (1987). The neighbor-joining method: A new method for reconstructing phylogenetic trees. Mol. Biol. Evol..

[28] Zuckerkandl E., Pauling L. (1965). Molecules as documents of evolutionary history. J. Theor. Biol..

[29] Tamura K., Peterson D., Peterson N., Stecher G., Nei M., Kumar S. (2011). MEGA5: Molecular evolutionary genetics analysis using maximum likelihood, evolutionary distance, and maximum parsimony methods. Mol. Biol. Evol..

[30] Madeira F., Park Y.M., Lee J., Buso N., Gur T., Madhusoodanan N., Basutkar P., Tivey A.R.N., Potter S.C., Finn R.D. (2019). The EMBL-EBI search and sequence analysis tools APIs in 2019. Nucleic Acids Res..

[31] Chow C.N., Lee T.Y., Hung Y.C., Li G.Z., Tseng K.C., Liu Y.H., Kuo P.L., Zheng H.Q., Chang W.C. (2019). PlantPAN3.0: A new and updated resource for reconstructing transcriptional regulatory networks from ChIP-seq experiments in plants. Nucleic Acids Res..

[32] Zouine M., Maza E., Djari A., Lauvernier M., Frasse P., Smouni A., Pirrello J., Bouzayen M. (2017). TomExpress, a unified tomato RNA-Seq platform for visualization of expression data, clustering and correlation networks. Plant J..

[33] Smeets K., Ruytinx J., Semane B., Van Belleghem F., Remans T., Van Sanden S., Vangronsveld J., Cuypers A. (2008). Cadmium-induced transcriptional and enzymatic alterations related to oxidative stress. Environ. Exp. Bot..

[34] Huang H., Ullah F., Zhou D.X., Yi M., Zhao Y. (2019). Mechanisms of ROS regulation of plant development and stress responses. Front. Plant Sci..

[35] Drążkiewicz M., Skórzyńska-Polit E., Krupa Z. (2007). The redox state and activity of superoxide dismutase classes in *Arabidopsis thaliana* under cadmium or copper stress. Chemosphere.

[36] DalCorso G., Manara A., Furini A. (2013). An overview of heavy metal challenge in plants: From roots to shoots. Metallomics.

[37] Pilon M., Ravet K., Tapken W. (2011). The biogenesis and physiological function of chloroplast superoxide dismutases. Biochim. Biophys. Acta.

[38] Huybrechts M., Cuypers A., Deckers J., Iven V., Vandionant S., Jozefczak M., Hendrix S. (2019). Cadmium and plant development: An agony from seed to seed. Int. J. Mol. Sci..

[39] Yan H., Jia H., Chen X., Hao L., An H., Guo X. (2014). The cotton WRKY transcription factor GhWRKY17 functions in drought and salt stress in transgenic *Nicotiana benthamiana* through ABA signaling and the modulation of reactive oxygen species production. Plant Cell Physiol..

[40] Barth O., Vogt S., Uhlemann R., Zschiesche W., Humbeck K. (2009). Stress induced and nuclear localized HIPP26 from *Arabidopsis thaliana* interacts via its heavy metal associated domain with the drought stress related zinc finger transcription factor ATHB29. Plant Mol. Biol..

[41] Graham H.C., Alison G.R., Susan J., Kumar P., Kalyandurg P.B., Gil J.F., Savenkov E.I., Hemsley P.A., Torrance L. (2018). Potato Mop-Top Virus Co-Opts the Stress Sensor HIPP26 for Long-Distance Movement. Plant Physiol..

[42] Murashige T., Skoog F. (1962). A revised medium for rapid growth and bio assays with tobacco tissue cultures. Physiol. Plant..

[43] Hoagland D.R., Arnon D.I. (1950). The water-culture method for growing plants without soil. Circ. CA. Agric. Exp. Stn..

[44] Fields S., Song O. (1989). A novel genetic system to detect protein-protein interactions. Nature.

[45] Chien C.T., Bartel P.L., Sternglanz R., Fields S. (1991). The two-hybrid system: A method to identify and clone genes for proteins that interact with a protein of interest. Proc. Natl. Acad. Sci. USA.

[46] Zhang X., Henriques R., Lin S.S., Niu Q.W., Chua N.H. (2006). Agrobacterium-mediated transformation of *Arabidopsis thaliana* using the floral dip method. Nat. Protoc..

[47] Ramakers C., Ruijter J.M., Lekanne Deprez R.H., Moorman A.F.M. (2003). Assumption-free analysis of quantitative real-time polymerase chain reaction (PCR) data. Neurosci. Lett..

[48] Livak K.J., Schmittgen T.D. (2001). Analysis of relative gene expression data using real time quantitative PCR and the 2^−ΔΔCT^ method. Methods.

[49] Rao M.V., Davis K.R. (1999). Ozone-induced cell death occurs via two distinct mechanisms in Arabidopsis: The role of salicylic acid. Plant J..

