# Supplementary material for: The Tomato Metallocarboxypeptidase Inhibitor I, which Interacts with a Heavy Metal-Associated Isoprenylated Protein, Is Implicated in Plant Response to Cadmium
Source: Molecules. 2020 Feb 6;25(3):700. doi: 10.3390/molecules25030700 (PMC7038206; doi:10.3390/molecules25030700)
Supplement: Supplementary file 1 [file molecules-25-00700-s001.pdf]

```
SlHIPP26      MGVLDHISDMFDCSSEHSKHKRRKQLQTVEIKVKMDCEGCERKVRRSVEGMKGVSSVTIE      60
AtHIPP26      MGVLDHVSEMFDCSHGHK-IKKRKQLQTVEIKVKMDCEGCERKVRRSVEGMKGVSSVTLE      59
              ******:*:*****  *.  *:************************************:*

SlHIPP26      PKQHKLTVVGYVDPEKVVSRVAHRTGKKAEIWPYVPYDVVAHPYAQGVYDKKAPAGYVRR      120
AtHIPP26      PKAHKVTVVGYVDPNKVVARMSHRTGKKVELWPYVPYDVVAHPYAAGVYDKKAPSGYVRR      119
              ** **:********:***:*::******.*:************** ********:*****

SlHIPP26      D-DFQTNQLARASSTEVRYTTAFSDENPAACVVM      153
AtHIPP26      VDDPGVSQLARASSTEVRYTTAFSDENPAACVVM      153
                *  ..***************************
```

Figure 1. Sequence alignment of HIPP26 proteins of tomato and *Arabidopsis* (At4G38580) obtained by CLUSTAL Omega (https://www.ebi.ac.uk/Tools/msa/clustalo/). The consensus symbols: "*" identical residues; ":" residues with strongly similar properties; "." residues with weakly similar properties.

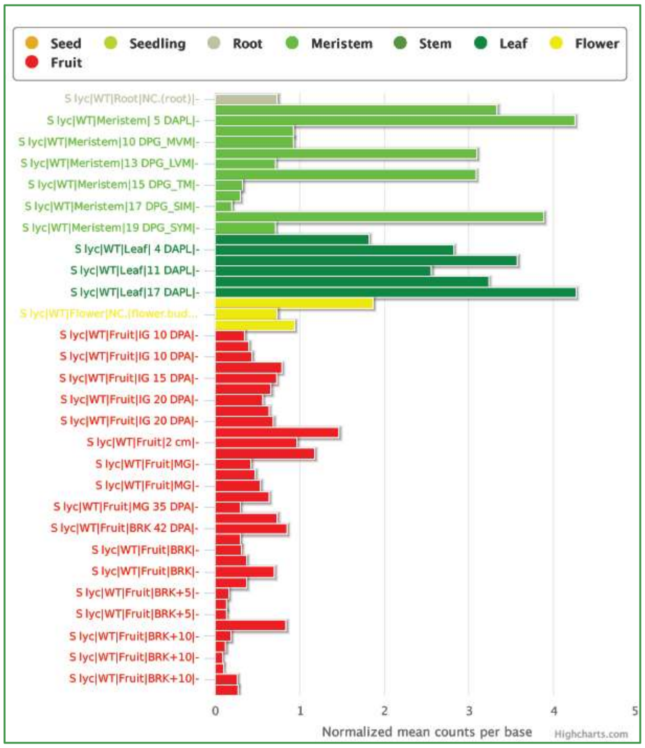

Figure 2. Expression of SlHIPP26 gene in organs of various *S. lycopersicum* cultivars.

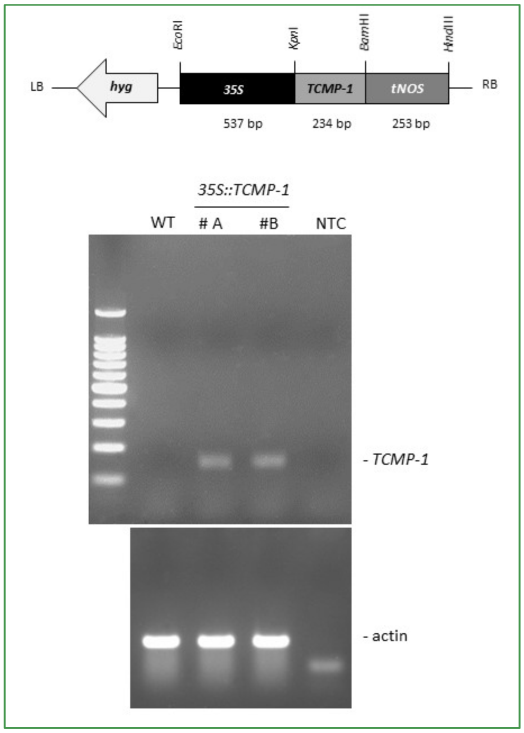

Figure 3. (Upper) Schematic drawing of *35S::TCMP-1* gene construct cloned into the T-DNA of the pCAMBIA 1200 vector. (Lower) RT-PCR analysis of TCMP-1 and actin gene expression conducted on WT and transgenic lines. NTC, not template control.

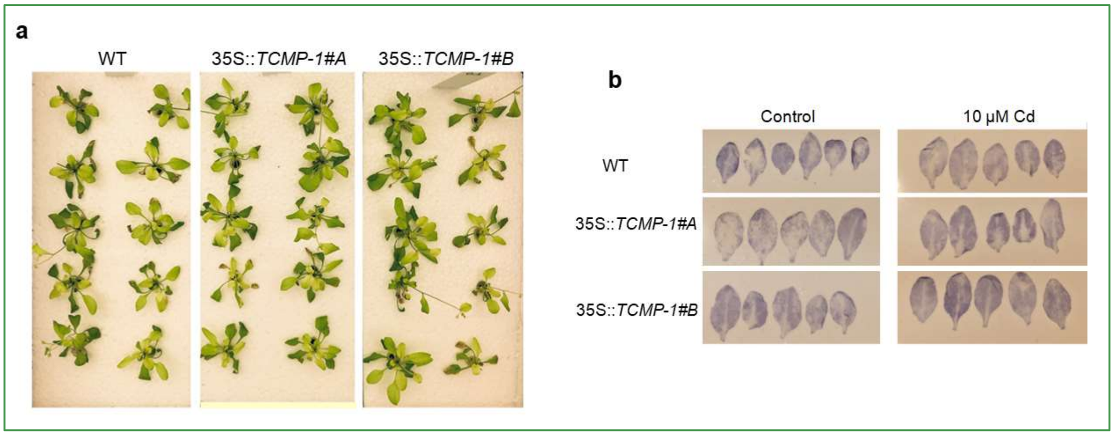

Figure 4. (a) Shoots of WT, *35S::TCMP-1#A* and *#B* plants after 10 days of hydroponic cultivation in the presence of 10 µM $CdSO_4$. **(b)** NBT staining to detect O^{2-} in the intact leaves of plants grown for 10 day in hydroponic Hoagland solution containing 10 µM $CdSO_4$.

Table 1. List of primers used in this work.

Gene	Application	Sequence (5′ →3′)
SlActin (Solyc11g005330.2.1)	Real-time RT-PCR	F: TTCAAAGGGCGAGTACGACGAG
		R: CAGCAGACCCGACTTCACTTTT
SlTCMP-1 (Solyc07g007250.4.1)	Real-time RT-PCR	F: GCTGCTCAAGATGTGATGGC
		R: TCCAACAGGCCTGACAGAAC
NtActin (XM_016628756.1)	Real-time RT-PCR	F: ATCCCAGTTGCTGACAATTC
		R: 5GACCCGCCATACTGGTGTGAT
NtMCPIa (AB518288.1)	Real-time RT-PCR	F: TAGGGACGATTGCTCTAAA
		R: ACTCTGTTAATACGAGGTAA
NtMCPIb (AB518289.1)	Real-time RT-PCR	F: CAGGGATGATTGCTCTAGT
		R: ACTTTGTTAATACGAGGTTT
AtUBI10 (At4g05320)	Real-time RT-PCR	F: AGGACAAGGAAGGTATTCCTC
		R: CTCCTTCTGGATGTTGTAGTC
AtHIPP26 (At4g38580)	Real-time RT-PCR	F: GTCGAGCTCTGGCCATATGT
		R: TACACCACTGCATTTAGCGAC
AtFSD1 (At4g25100)	Real-time RT-PCR	F: AGTTCTGCATCACCGAAGTCT
		R: ACAAGCAAAATGAAGAACTCTGT
AtFSD2 (At5g51100)	Real-time RT-PCR	F: TGTTATAGTGAAGACGCCCAAT
		R: AAAATCCAGATAGTAAGCGTGC
AtCSD1 (At1g08830)	Real-time RT-PCR	F: AAGAAGAGATTGATGTAATAAGGA
		R: CCACACTAAGCTCATAGGTTTT
AtCSD2 (At2g28190)	Real-time RT-PCR	F: GGCCATGAGCTTAGTCTGAC
		R: ACTTGCTTAGCCTCTGACTTA
SlTCMP-1 (37aa) (NM_001247005.3)	Y2H	F: CATGGAGGCCGAATTCCAGCAATATGATCCAGTTTGTCAC
		R: GCAGGTCGACGGATCCAACATAGGGCCCACATGTCC
SlHIPP26 (152 aa) (Solyc01g111600)	Y2H	F: GGAGGCCAGTGAATTCGGTGTTCTTGATCACATATCTGAC
		R: CGAGCTCGATGGATCCCATGACCACACAGGCTGC
